# High tetramethylpyrazine production by the endophytic bacterial *Bacillus subtilis* isolated from the traditional medicinal plant *Ligusticum chuanxiong* Hort.

**DOI:** 10.1186/s13568-018-0721-1

**Published:** 2018-12-18

**Authors:** D. D. Yin, M. Yang, Y. L. Wang, D. K. Yin, H. K. Liu, M. Zhou, W. Li, R. Chen, S. S. Jiang, M. F. Ou, F. Xu

**Affiliations:** 1 College of Pharmacy, Anhui University of Chinese Medicine, Hefei, 230012 Anhui China; 2 Laboratory of Chinese Medicinal Formula of Anhui Province, Hefei, 230012 Anhui China

**Keywords:** Rhizoma Chuanxiong, Tetramethylpyrazine, Endophytes, *Bacillus subtilis*

## Abstract

Tetramethylpyrazine (TMP) with significant protective effects on cardiovascular is the active ingredient of traditional Chinese medicine Rhizoma Chuanxiong (RC). However, many studies have reported the low content of TMP in RC. The endophytes of medicinal plants have the biosynthetic potential to produce the same or similar active metabolites as the host, while few reports were conducted to explore the endophytic bacteria of *Ligusticum chuanxiong* Hort. and its productive capacity for the important ingredient TMP. The present paper focuses on the isolation and identification of TMP producing endophytic bacteria from RC. In this study, the endophytic bacteria were isolated from the rhizome of *Ligusticum chuanxiong* Hort. (Umbelliferae). Yeast extract peptone glucose medium (YP) was used for fermentation medium (37 °C, 220 rpm agitation, 144 h). GC and GC/MS were performed to determine and verify the product, the fermentation characteristics were investigated. Morphological observation, physiological and biochemical indexes combining with 16S rRNA sequence analysis were carried out to identify the endophytic bacteria. As a result, five strains of endophytic *Bacillus subtilis* were firstly isolated and identified from RC, named as LB3, LB3-2-1, LB6-2, LB4, LB5 respectively. All five strains of endophytic *B. subtilis* produced TMP, while LB5 had the highest production of 10.69 g/L at the 144 h fermentation. This work demonstrates the fact that the endophytic *B. subtilis* of RC can produce a high level of TMP, indicating the endophytic *B. subtilis* might play a role in the accumulation of TMP during the growth period of RC.

## Introduction

*Ligusticum chuanxiong* Hort. (LC) is one of the herbaceous perennial plants which belongs to the Umbelliferae family and the dry rhizome of LC is the only source of Rhizoma Chuanxiong (RC). RC is famous for its effect of promoting blood circulation and removing blood stasis. It has been widely and extensively applied for many years to treat some cardiovascular pathological settings such as atherosclerosis, ischemic stroke, migraine and coronary heart disease with good efficacy (Yang et al. 2016; Ran et al. 2011; Li et al. 2012, 2013; Wang et al. 2013; Zhang and Liu 2016).

Clinical practice and pharmacological studies indicated that TMP (Ligustrazine) is the vital and representative component of RC. The function of antiplatelet aggregation plays a critical role in the pathogenesis of atherothrombosis (Wang et al. 1999). TMP significantly improved neurological function at 7 d and 14 d after ischemia (Lin et al. 2015) and provided neuroprotection against ischemic brain injury (Kao et al. 2006). Research shows TMP can promote neuronal differentiation through epigenetic regulation of Topoisomerase IIβ (TopoIIβ) (Yan et al. 2014). Its promote neural progenitor/precursor cells migration (Kong et al. 2016) and protects neurons from oxygen–glucose deprivation-induced death (Shao et al. 2017). TMP also protects bone marrow-derived mesenchymal stem cells against H_2_O_2_-induced apoptosis by regulating the PI3K/Akt and ERK1/2 signalling pathways (Fang et al. 2017; Li et al. 2017).

However, with the development of analytical techniques and the application of online mass spectrometry (LC/MS/MS, GC–MS, UPLC–Q-TOF-MS), more and more studies have shown that the content of TMP (range from 0. 60 to 11.75 μg/g) in RC is very low (Hong et al. 2015; Zhu et al. 2011; Song and Xu 2013). Some studies even can not detect it with the lower detection limit (0.1 μg/g) by HPLC (Li et al. 2001; Guo et al. 2016). As the active ingredient in RC, the trace of TMP partly hinders the clinical efficacy. As a consequence, it is hard to meet clinical applications by extracting TMP from RC. Currently, Chemical synthesis is the staple and convenient way to prepare TMP for medical use. However, this is harmful to the environment (Cenker et al. 1964; Miriam et al. 1995).

Plant endophytes are microorganisms that live in plant tissues without causing symptoms of the disease, and they are essential components of plant microbiomes (Porras-Alfaro and Bayman 2011). Endophytes provide a broad variety of bioactive secondary metabolites include alkaloids, cytochalasins, polyketides, terpenoids, flavonoids and steroids (Guo et al. 2008; Strobel and Daisy 2003). It has been reported that *B. subtilis* catabolizes glucose to produce 3-hydroxy-2-butanone (HB) which, as a precursor to TMP, is further converted to TMP with the stimulating effect participation of free ammonium ions (Zhu et al. 2010; Zhu and Xu 2010a, b) In this biochemical process, HB can be reversibly transformed into its reductive state 2.3-butanediol (BD), which was catalyzed by BD dehydrogenase (BDH). So far, a total of five strains of *B. subtilis* have been studied for the microbial production of TMP. They are, respectively, *B. subtilis* BS2 and it’s mutant strains *B. subtilis* BSA, *B. subtilis* RX3-17 isolated from soil (Xiao et al. 2006), *B. subtilis* CCTCC M 208157 isolated from a Chinese Maotai-flavor Daqu (Zhu et al. 2010) and *B. subtilis* IFO 3013 isolated from *natto* (Besson et al. 1997; Larroche et al. 1999). It is unknown whether there are endophytic bacteria in RC possessing the productivity of TMP and what fermentation characteristics they are.

In this paper, we aimed at screening and isolating endophytic bacteria from RC and investigating secondary metabolites of the endophytes. These findings demonstrate that the newly isolated strains of *B. subtilis* from RC were capable of producing high levels of TMP. Among them, *B. subtilis* LB5 was the best TMP producer with a production of 10.69 g/L.

## Materials and methods

### Plant materials, chemicals, and reagents

Healthy RC plants were collected from Pengzhou district of Sichuan Province on May 13, 2017. The reference standard of TMP, 3-hydroxy-2-butanone (HB) were purchased from Solarbio Life Science (> 98%, Beijing, China). Dichloromethane (HPLC grade) was obtained from Sinopharm Chemical Reagent (Shanghai, China). All other chemicals used in this investigation were of analytical grade available commercially.

### Isolation and cultivation of endophytes

The fresh rhizome of RC was washed thoroughly using running tap water. The concentration (1–15%) and sterilization time (1–6 min) of sodium hypochlorite were tested to ensure the complete surface sterilization of the rhizome. Removed the epidermis, sections of the rhizome of 1 cm long were surface-sterilized followed by 75% ethanol for 5 min, 5% NaClO solution for 3 min. Surface-sterilizing agents were subsequently removed by rinsing the plant material six times with sterile distilled water. After the sample dried, it was ground in 5 mL sterile distilled water under aseptic conditions. Then 50 μL were plated on the nutrient agar (5.0 g peptone, 3.0 g beef extract, 2.5 g glucose, 1.0 g yeast extract, 18 g agar, 1000 mL distilled water, pH 7.2) at 37 °C for 48 h. According to the colony morphology and colour of *B. subtilis*, possible single colonies were picked and incubated in nutrient agar medium. Streak plate method was used to purify them till a single strain. All experiments were performed in triplicates.

### Shake flask fermentation

The strains were inoculated aerobically on a nutrient agar medium for 48 h at 37 °C. Then, they were inoculated onto 50 mL nutrient broth in 300 mL shaking flask for 48 h (37 °C, 220 rpm). The nutrient broth was used as the seed culture. Then the seed culture was inoculated (6%, v/v) into YP medium (100 g, glucose 30 g peptone, 10 g yeast extract, 30 g (NH_4_)_2_HPO_4_, 1000 mL distilled water, pH 7.20) as the fermentation medium (50 mL medium in a 300-mL Erlenmeyer flask, 37 °C, 220 rpm, 144 h).

### GC and GC/MS analysis of products

The fermentation broth was centrifuged at 13,000 rpm for 10 min (4 °C), then the supernatant was extracted by dichloromethane with the sample and dichloromethane volume ratio of 1:3. GC system (Agilent 7890B, USA) equipped with flame ionization detector (FID) and HP-5 MS capillary column (30 m longness, 0.25 mm inside diameter, 0.25 μm film thickness) was used. We took a split injection with a split ratio of 19:1 and the injection volume was 2 μL. The injector temperature was 230 °C, and the detector temperature was 280 °C. The column oven was kept constant at 50 °C for 6 min, and then programmed to 210 °C with a temperature increase of 12 °C/min. TMP standard and HB standard were dissolved in methanol to prepare a stock standard (2, 1, 0.8, 0.4, 0.2, 0.1, 0.05 mg/mL). Standard curves were constructed, and validation of quantitative method was performed regarding precision, stability, repeatability and extraction recovery. An aliquot (2 μL) of each extract was subjected to GC analysis.

Products in the fermentation broth were further identified by 456-GC system equipped with SCION single quadrupole mass spectrometer (Bruker Corp., USA). High purity nitrogen and high purity helium were used as the carrier gas. The flow rate was 1 mL/min. The injection volume was 2 μL while the split ratio was 10:1. Programmed temperature increase conditions of column oven were same as GC condition. Mass spectra in the electron impact mode were generated at 70 eV and full scan mode in the range of 50–500. The temperature of the ion source, transmission line and quadrupole were 220 °C, 260 °C and 40 °C, respectively. The NIST 11. L was used as mass spectral search standard library.

### Study on fermentation characteristics

Samples were withdrawn from the fermentation medium every 12 h to explore the fermentation characteristics of high production TMP strain. Cell growth was measured by standard plate count method (Roy et al. 2018). The appropriate dilution concentration was chosen, and each sample was run in parallel for three groups to detect the number of viable cells. The fermentation broth was harvested by centrifugation at the speed of 13,000 rpm for 10 min (4 °C), the supernatant was assayed for products by GC. The pH of the fermentation broth was estimated by pH meter. The content of residual sugar in fermentation broth was determined by Microplate System (Thermo, USA) at the wavelength of 550 nm with DNS method. DNS reagents should be configured 1 week in advance and put on the cryogenic (4 °C), dark place.

### Identification of isolated strains

Microscopic slides were prepared, stained using Gram stain kit and Spore staining kit respectively. Isolated strains were tentatively identified and selected according to key physiological and biochemical characteristics of *B. subtilis* (Buchanan and Gibbons 1974). The catalase reaction, Methyl red reaction and Voges–Proskauer (V–P) test were carried out. The utilization of carbohydrate (including d-glucose, d-lactose and citrate) was performed. Decomposition experiments of macromolecular compounds were explored, such as hydrolysis of gelatin and starch, assay of catalase. The production of indole and nitrate reduction were also tested.

The total genomic bacterial DNA was extracted by the CTAB method with bacterial genomic DNA extraction kit (Basheer et al. 2018; Stachelska 2017). The endophytic bacterial were identified by amplifying the 16S ribosomal region of isolated DNA using universal bacterial primers. The forward primer was 5′AGAGTTTGATCCTGGCTCAG-3′, and the reverse primer was 5′CGGCTACCTTGTTA CGAC3′. The amplified PCR product was checked on 1% agarose gel electrophoresis and visualised on the gel imaging system. After the PCR product was sequenced, the National Center for Biotechnology Information (http://www.ncbi.nlm.nih.gov) was accessed, and BLAST program was used to align the sequence with that from the GenBank database for homology analysis. A phylogenetic tree was constructed and subsequently analysed for evolutionary distances in MEGA 6.0 software.

## Results

### Isolation and Identification of endophytes

We had isolated 54 pure strains of endophytic bacteria from the fresh rhizome of RC. After GC analysis of their corresponding fermentation samples, there were 5 strains shown to produce TMP. Therefore, we identified these 5 endophytic bacteria. The five pure strains of endophytic bacteria were suspected of *B. subtilis.* The isolated strains on the nutrient agar medium were off-white, rod-shaped. It was Gram-positive and Spore-positive. All of the five endophytic bacteria were methyl red test-negative, V–P test-positive. Citrate utilised but not malonate. At the same time, it made use of d-glucose without producing gas and d-xylose to produce acid. The results of nitrate reduction showed that five endophytic bacteria reduced nitrate to nitrite without indoles generated. Also, the five isolated endophytic bacteria hydrolysed macromolecular substances such as gelatin, starch. The results of critical physiological and biochemical tests (shown in Table 1) further verified that the five endophytic bacteria could be *B. subtilis*. The five strains were sequentially named as LB3, LB3-2-1, LB4, LB4, LB6-2.

**Table 1:** The physiological and biochemical characteristics of five endophytic bacteria

Project	LB3	LB3-2-1	LB6-2	LB4	LB5
catalase reaction	+	+	+	+	+
Voges–Proskauer reaction	+	+	+	+	+
Methyl red reaction	−	−	−	−	−
Nitrate reduction	+	+	+	+	+
d-glucose utilization	+	+	+	+	+
d-lactose utilization	+	+	+	+	+
Citrate utilization	+	+	+	+	+
Hydrolysis of gelatin	+	+	+	+	+
Hydrolysis of starch	+	+	+	+	+
Indole production	−	−	−	−	−

“+” represents the test results were positive; “−” represents the test results were negative

**Fig. 1:**
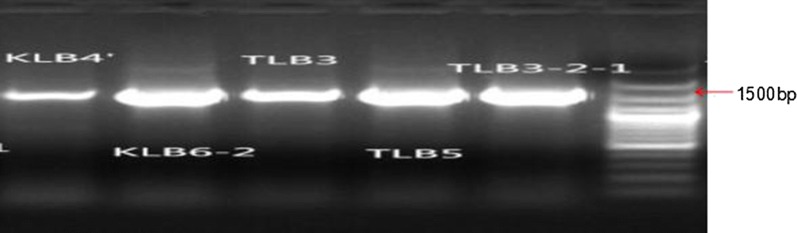
The agarose electrophoresis of five endogenetic strains 16S rRNA gene products. K and T represent the kinds of medium, K is the abbreviation of King‘s B medium; T is the abbreviation of Tryptic soy agar medium. From left to right is K LB4, KLB6-2, TLB3, T LB5, TLB3-2-1, and maker respectively

**Fig. 2:**
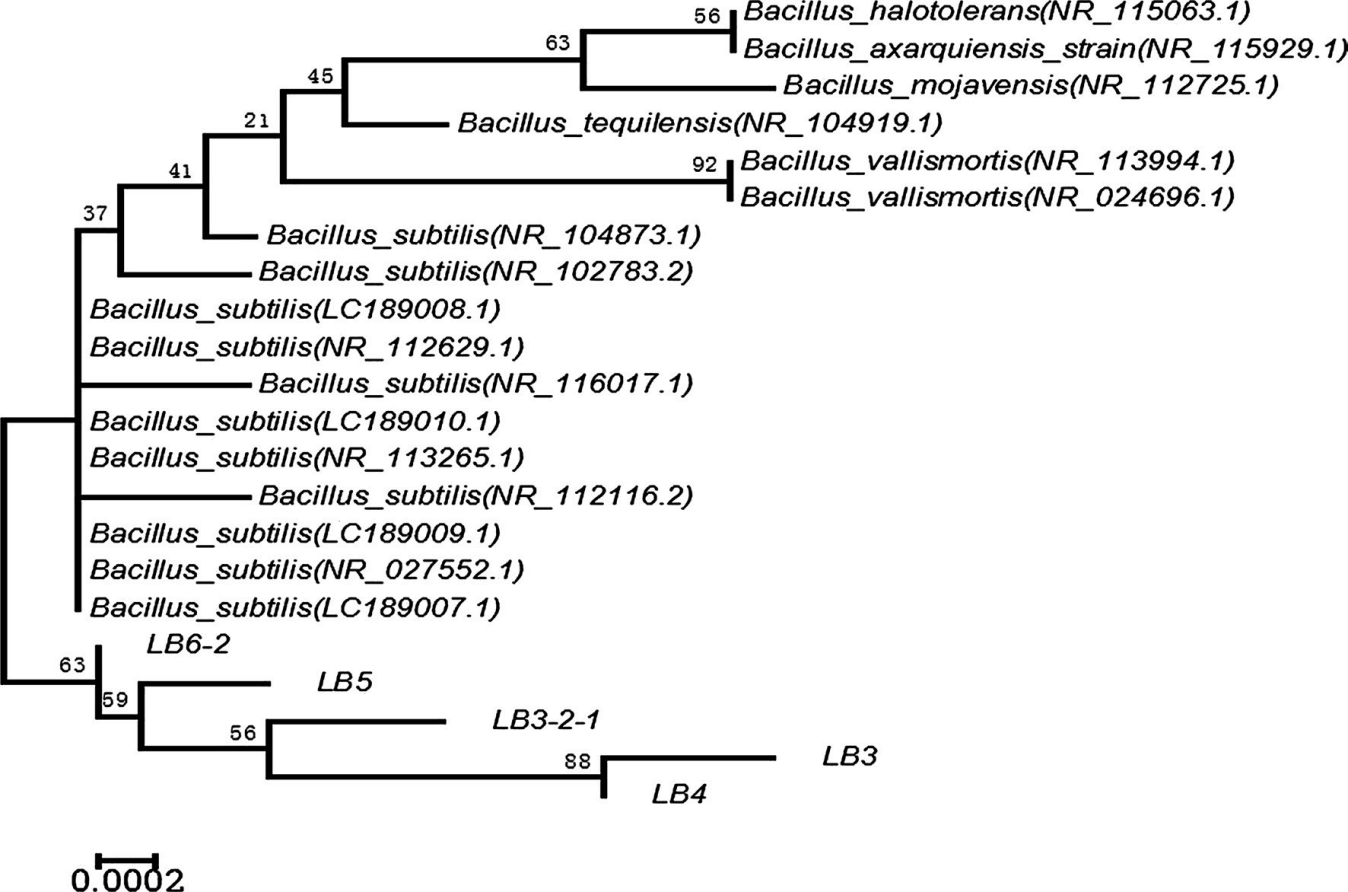
The neighbour-joining phylogenetic tree of five strains of endophytic bacteria. The representative strains that have been identified as *B. subtilis* from Genebank were selected to construct the neighbour-joining phylogenetic tree by MEGA 6.0 software. The values of confidence levels obtained from 1000 bootstrap replicates above 50% are shown on branches nodes. Scale bar represents 0.0002 substitutions per nucleotide position

The alignment of 16S rRNA sequence obtained from LB3, LB3-2-1, LB6-2, LB4, LB5 showed 99% homology with the corresponding gene sequences of *B. subtilis*. The agarose gel bands after 16S rRNA PCR amplified gene fragments were shown in Fig. 1. The phylogenetic tree based on 16S rRNA gene sequence showing the taxonomical position of LB3, LB3-2-1, LB4, LB5, LB6-2 (Fig. 2). The molecular identification indicated that all five strains of RC endophytic bacteria isolated from RC were *B. subtilis.* The 16S rRNA sequence of the five strains have been deposited to the GenBank under accession numbers MH197362 (LB3), MH197360 (LB3-2-1), MH197363 (LB4), MH197364 (LB5), MH197361 (LB6-2). *B. subtilis* LB5 was deposited in GuangDong Culture Collection Center (GDMCC 1.1490).

### GC and GC/MS analysis of TMP and HB

**Fig. 3:**
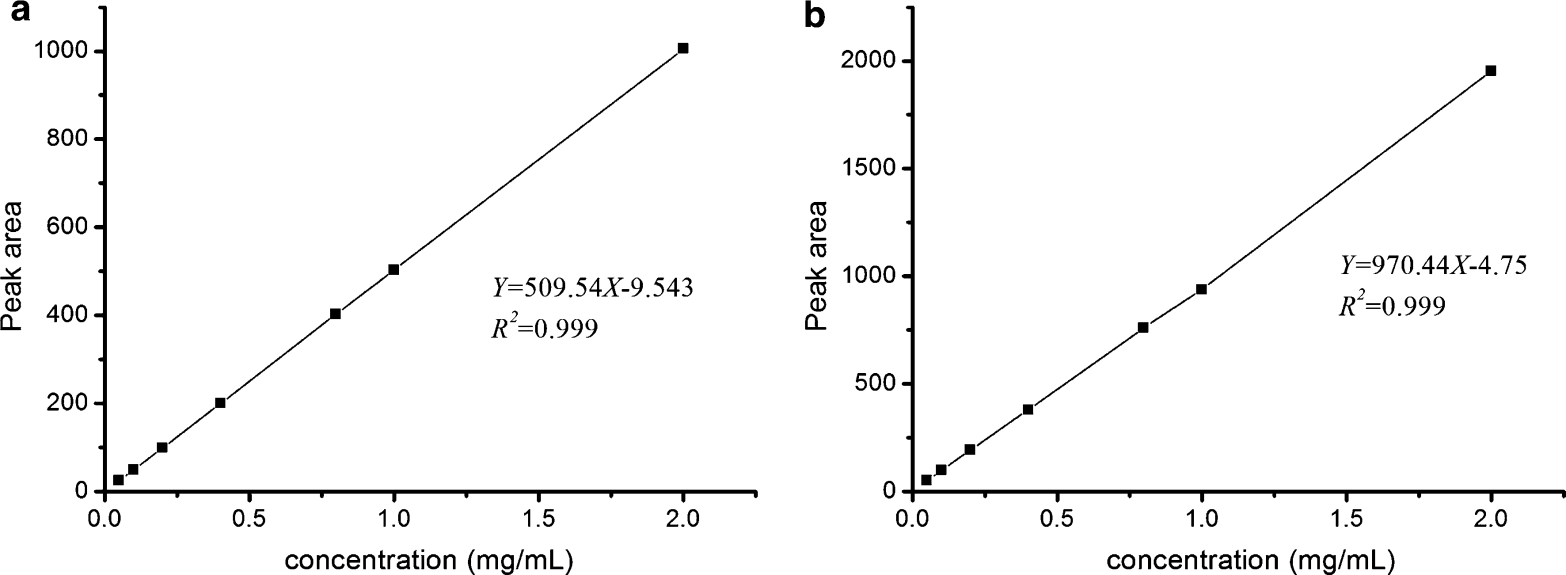
Standard curves of authentic compounds. **a** standard curve of TMP, **b** standard curve of HB

**Fig. 4:**
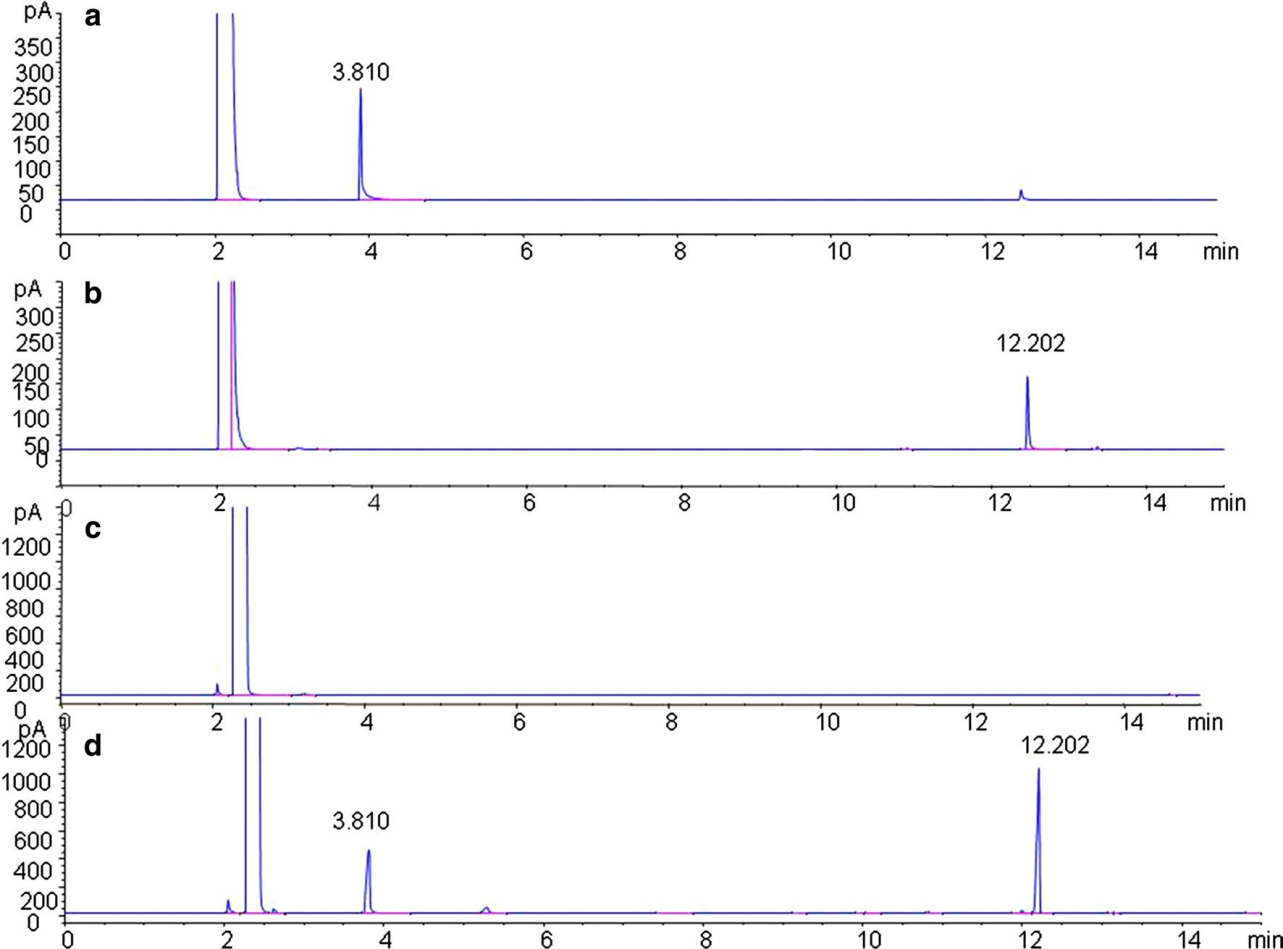
GC chromatograms of the fermentation broth sample. **a** chromatograms of the authentic compounds TMP, **b** chromatograms of the authentic compounds HB, **c** fermentation broth samples of the blank group, **d** fermentation broth samples of LB5

We adopted the standard external method to establish the standard curves of TMP and HB (Fig. 3). The regression equation of TMP and HB were *Y *= 509.54*x* − 9.543 (*R*^*2*^= 0.999), *Y *= 970.44*x* − 4.75 (*R*^*2*^= 0.999) respectively, showing good linearity in the concentration ranges tested. Furthermore, the validation studies provided good precision with the relative standard deviation (RSD) of TMP and HB were 0.75% and 0.78%, respectively. In the stability test of time (0 h, 2 h, 4 h, 6 h) and storage temperature (25 °C, 4 °C, − 20 °C), the RSDs for TMP were 1.23%, 1.33% and for HB 1.09%, 1.45%. As for the repeatability, the RSD of TMP and HB were 0.92% and 1.59%. Furthermore, when the 2 standards were added to the samples with known TMP and HB content, the RSD of recovery test for TMP and HB were 0.92% and 0.83%. These data justified that the GC method was applicable for the analysis of TMP and HB in the fermentation broth. As shown in Fig. 4, the compounds in the fermentation broth were assayed under the optimised chromatographic condition. The results showed that all five strains of endophytic bacteria from RC produced high levels of TMP (Table 2). Moreover, LB5 had the highest production of TMP at the end of fermentation.

**Table 2:** The final yield of TTMP and HB of endophytic B. subtilis

	LB3	LB3-2-1	LB6-2	LB4	LB5
TMP (g/L)	9.536	8.505	8.181	10.24	10.69
HB (g/L)	36.70	30.26	44.46	43.16	51.31

Data were obtained after 144 h fermentation by GC analysis, the conditions of fermentation and GC analysis were shown in the text

**Fig. 5:**
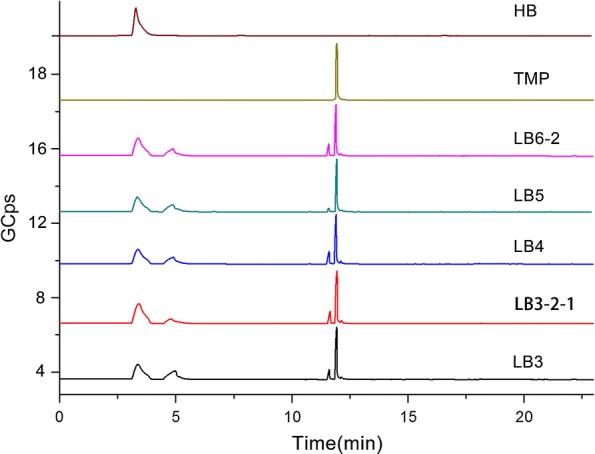
Total ion current chromatogram of five strains of endophytic bacteria isolated from RC. The spectra of samples matched well with authentic compounds of HB and TMP, 2,3-BD and 1,1-DAH were identified from MS data library. The retention time was 3.888 min for HB, 4.888 min for 2,3-BD, 11.608 min for 1,1-DAH and 11.898 min for TMP

**Fig. 6:**
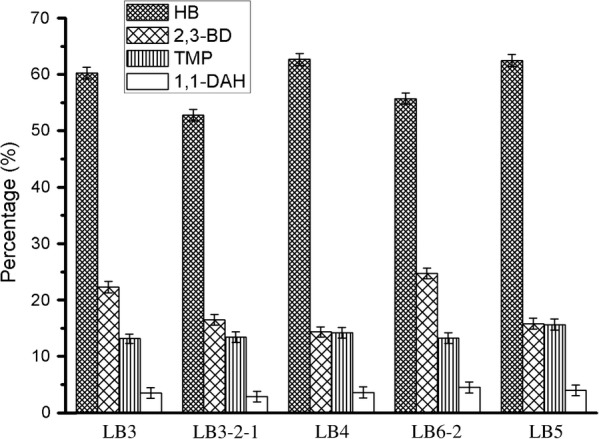
The relative abundance of the five endophytes metabolites in the dichloromethane extraction site. Data were obtained after 72 h fermentation by GC/MS analysis. All samples were measured in triplicates; standard deviations were always found less than 10% in all experiments

Additionally, the TMP and HB products were determined by GC/MS; 2,3-butanediol (2,3-BD) and 1,1-diallylhydrazine (1,1-DAH) were identified as well (Fig. 5). After mass spectrometry scan, the mass spectra of each peak in the total ion chromatogram obtained were determined through the mass spectrometry data system retrieval of NIST 11.L, combined with manual spectral analysis, the base peak, mass-to-charge ratio and relative abundance and other aspects of an intuitive comparison. A total of 4 chromatographic peaks were identified as TMP, HB, 2,3-BD and 1,1-DAH respectively. Furthermore, the relative percentage of each component was analysed using peak area normalisation method. The relative content of TMP, HB, 2,3-BD and 1,1-DAH of the five strains were correspondingly shown in Fig. 6. We chose the 72-h fermentation broth as the analysis sample of GC–MS. At that time, the endophytic *B. subtilis* catabolized glucose under the participation of *α*-acetolactate synthase (ALS) and *α*-acetolactate decarboxylase (ALDC), to produce a large number of primary metabolites such as HB and 2,3-BD (Larroche et al. 1999; Besson et al. 1997). The reversible transformation between HB and its reductive state (2,3-BD) was catalyzed by BD dehydrogenase (BDH) (Meng et al. 2015; Renna et al. 1993). However, the conversion of HB to TMP in the fermentation broth at 72 h was not very obvious.

Furthermore, 1,1-DAH was found in the metabolites of endophytic bacteria isolated from RC. 1,1-DAH, a derivative of hydrazine, had good solubility in water. According to previous studies, 1,1-DAH was an extensive co-product from chemical synthesis of allyl hydrazine with the reaction between allyl bromide and hydrazine monohydrate at 70 °C for 1 h (Pohako et al. 2008). It was the first time that 1,1-DAH was found in metabolites of microbial. The result obtained was to some extent different with the previous report, probably due to the diverse fermentation property of the microbe used in the investigation. However, the identification results of 1,1-DAH need further identification means to validate it. Although the formation mechanism of TMP in fermentation system is still controversial at present, the combination of nitrogen atoms in the organic state with HB is an important step in the biosynthesis of TMP. We speculate that 1,1-DAH was a by-product during that convertion. The formation of 1,1-DAH in the fermentation broth was partly due to the action of enzymes catalysing ammonium ions to become organic nitrogen atom.

### Study on fermentation characteristics of LB5

**Fig. 7:**
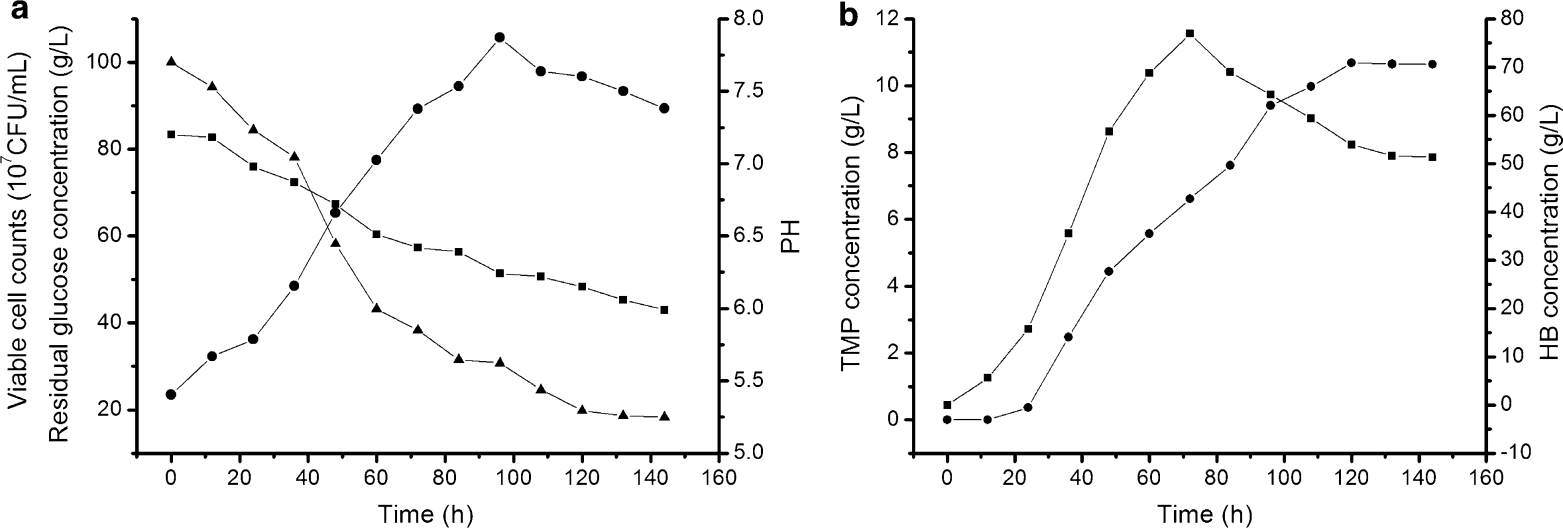
The fermentation process curve of LB5. Viable cells counts, residual glucose, PH value, the content of TMP and HB in the fermentation broth of LB5 were detected every 12 h a time till 144 h. **a** Black circle viable cells, black up-pointing triangle residual sugar content, black square PH value. **b** black square HB, black circle TMP

Of the five strains, LB5 have the highest yield of TMP. Therefore, we studied the flask fermentation characteristics of LB5. We measured viable cell counts, the residual glucose content and PH value of LB5 fermentation broth at different sampling points (Fig. 7a). Glucose (10% initial content) consumption was positively correlated with cell propagation of LB5, and the number of vible cells reached its maximum (105.7*10^7^ CFU/mL) at 96 h. The depletion of glucose was continuously underway, which was consistent with the previous reports (Zhu et al. 2010; Meng et al. 2015; Xiao et al. 2006). The decrease of PH value from initial 7.20 to terminal 5.99 indicated LB5 could produce acidic substances. Studies have shown that lactic acid is the main non-volatile by-product of glucose metabolism and regulation the pH of the medium had a significant effect on the accumulation of TMP (Xiao et al. 2006; Zhu and Xu 2010b).

As shown in Fig. 7b, the content of TMP and HB in fermentation broth were detected by GC. The maximum accumulation of HB was 76.93 g/L when LB5 was cultivated for 72 h. After that, as the precursor of TMP biosynthesis, there was a gradual decline in the concentration of HB as the increase of TMP. On the other hand, the conversion of 2,3-BD was an important factor affecting the level of HB, which in turn changed the production of TMP. TMP production reached its maximum 10.69 g/L when LB5 cultivated for 120 h. Without any stimulating strategy, LB5 naturally ferments in YP medium with an output of TMP 10.69 g/L and HB 51.31 g/L at the end of fermentation. These results indicated that the endophytic *B. subtilis* of LC were competent to produce TMP.

## Discussion

TMP was extracted from the Chinese medicine RC since 1976 (Beijing Institute of Pharmaceutical Industry 1977), it had been increasingly studied for its action on myocardial and cerebral infarction, and ligustrazine hydrochloride has been extensively used for coronary heart disease, myocardial and cerebral infarction many years. Whereas its content in RC is very low and the oral concentration of TMP in RC is lower than the clinically effective dose of TMP monomer (average dose 178 mg/days), TMP was reported to be the main active and representative ingredient of RC. It was a paradox.

What is interesting is TMP was initially found in the metabolites of *B. subtilis* isolated from Japanese traditional soy fermented food natto in 1962 (Kosuge and Kamiya 1962a) and TMP could accelerate the growth of *B. subtilis* (Kosuge and Kamiya 1962b). *B. subtilis* shows great productivity in the production of TMP. This is not only reflected in the ability to decompose a variety of sugar sources such as sucrose, fructose, glucose and cellulose, but also in the secretion of extracellular proteins. HB can be reversibly transformed into its reductive state 2.3-butanediol (BD), which was catalysed by BD dehydrogenase (BDH). By disrupting the 2,3-BD dehydrogenase gene (bdhA) of *B. subtilis*, the precursor HB accumulation could improve in the early stationary phase (Meng et al. 2015).

For the first time, we successfully isolated five endogenetic *B. subtilis* from RC. After fermentation, GC analysis results showed that all the five strains producing a high yield of TMP. Repeated experimental results showed that the high-yield TMP genetic characteristics of the five were stable. It is worth noting that we had never adjusted strategies such as applying the exogenous precursor, adding nutrients and free ammonium ion, controlling of PH during the fermentation process, but achieved higher yields compared with previous reports. Consequently, our results indicated that the five stains of endogenous *B. subtilis* of RC have a high ability to catabolize glucose to produce HB and TMP. However, TMP is the main active ingredient in RC, but its content is very low. High yield of TMP by five endogenous *B. subtilis* is in sharp contrast with the trace TMP in RC. The role of RC and endophytic *B. subtilis* in the accumulation process of TMP in RC is worth further study.
